# Study on the Performance of Coal Gangue-Loaded Hydroxyapatite (CG@HAP) for the Adsorption of Malachite Green

**DOI:** 10.3390/molecules29235649

**Published:** 2024-11-28

**Authors:** Junli Shao, Di Wu

**Affiliations:** 1 College of Mechanics and Engineering, Liaoning Technical University, Fuxin 123000, China; zidane668@163.com; 2 College of Science, Liaoning Technical University, Fuxin 123000, China

**Keywords:** coal gangue, loading, hydroxyapatite, adsorption, malachite green

## Abstract

In response to the issues of dye wastewater pollution and coal gangue accumulation, a novel adsorbent, coal gangue-loaded hydroxyapatite (CG@HAP) was prepared using coal gangue as the raw material for the adsorption of malachite green dye wastewater. Based on batch experiments, combined with adsorption kinetics and isotherm models, as well as XRF, FTIR, XRD, and SEM analysis, the characteristics of CG@HAP in adsorbing malachite green were investigated. The results show that CG@HAP can be prepared by adding 150 mL of 0.15 mol/L (NH_4_)_2_HPO_4_ solution and 150 mL of 0.25 mol/L CaCl_2_ solution to 10 g coal gangue under the condition of pH = 10, allowing it to stand at room temperature for 24 h. When the dosage of CG@HAP was 0.10 g and the adsorption time was 180 min, the adsorption removal rate and adsorption capacity of CG@HAP for 400 mg/L malachite green reached 92.62% and 370.49 mg/g, respectively. The adsorption of malachite green by CG@HAP followed the pseudo-second-order kinetic model and the Langmuir isotherm model. The adsorption of malachite green by CG@HAP was primarily governed by chemical reactions, adhering to the Langmuir monolayer adsorption principle. The maximum adsorption capacity of CG@HAP for malachite green was 386 mg/g. CG@HAP exhibited sustained and efficient dynamic adsorption of malachite green, maintaining a removal rate between 83.52% and 99.96%. CG@HAP proved to be an efficient adsorbent for malachite green, with great potential for application.

## 1. Introduction

With the rapid development of industries such as textiles and printing, the discharge of non-degradable synthetic dye wastewater has been increasing [1]. Most synthetic dyes are toxic and non-biodegradable [2]. Efficient treatment of dye wastewater has become one of the key challenges for achieving environmental sustainability [3]. Malachite green and other dyes belong to a class of non-degradable organic compounds, which, if retained in humans or other organisms for a long time, can leave highly toxic residues and potentially cause cancer. Traditional chemical and biological methods for treating dye wastewater often suffer from high costs, poor dye decolorization efficiency, and secondary pollution issues [4,5]. It has been reported that the adsorption method is the simplest and most effective technique for treating dye wastewater [6]. The main factor affecting the effectiveness of the adsorption method is the choice of adsorbent. Hydroxyapatite (HAP) is a type of apatite mineral known for its good biocompatibility, non-toxicity, and low cost [7,8]. Due to the ion-exchangeability of Ca(II) in the HAP structure, HAP has good adsorption properties for heavy metal ions and has been widely used in heavy metal adsorption fields [9]. For example, Kousalya G. et al. [10] synthesized nano-hydroxyapatite (n-HAp), an n-HAp/chitin composite, and an n-HAp/chitosan composite, with adsorption capacities for Fe(III) of 4.24, 5.80, and 6.75 mg/g, respectively. Chenglong Zou et al. [11] prepared HAP-functionalized magnetic rice husk biochar with a maximum adsorption capacity of 81.59 mg/g for Cu(II). Zou X. et al. [12] showed that porous nano-hydroxyapatite spheres can effectively adsorb and remove Pb(II), Cd(II), Cu(II), Ni(II), and Hg(II), with a maximum adsorption capacity of 254.90 mg/g. Many studies emphasize HAP’s practicality in heavy metal treatment. Additionally, HAP has a large specific surface area and abundant surface functional groups, making it suitable for the adsorption of organic compounds. Cleibson O. et al. [13] synthesized zinc(II)-modified hydroxyapatite, which exhibited a maximum adsorption capacity of 168.5 mg/L for tetracycline. Cheng Wang et al. [8] synthesized a porous carbon-loaded HAP composite adsorbent, achieving decolorization rates of 93.19% and 92.41% for sucrose juice and molten syrup, respectively. Manisha Sukhraj Kothari et al. [14] synthesized a carbide slag-based hydroxyapatite, which had an equilibrium adsorption capacity of 36 mg/g for methylene blue. Zaineb Mchich et al. [15] prepared HAP–polyaniline microcomposites, which exhibited adsorption of Orange G following a pseudo-second-order kinetic model and Freundlich model. Aghilas Brahmi et al. [16] synthesized porous hydroxyapatite–kaolinite polymer composites with an adsorption capacity of approximately 41 mg/g for Brilliant Green dye. These studies demonstrate that HAP has good adsorption performance for organic compounds such as tetracycline, syrup, and dyes. However, due to the high surface activity of HAP, it tends to aggregate under van der Waals forces, resulting in poor flowability and decreased adsorption performance. To address this issue, many studies have combined HAP with other materials to create composite adsorbents, preventing HAP agglomeration and improving adsorption performance. Therefore, finding a large, inexpensive material to combine with HAP to prevent aggregation has become a research hotspot.

Coal gangue, as a solid waste from mining areas, has the advantages of large accumulation and low cost. Converting coal gangue into a new adsorbent material for wastewater adsorption and purification is an active strategy for resource recycling and reducing waste accumulation [17]. Studies have shown that coal gangue can be used as an adsorbent to treat wastewater, but its adsorption capacity is limited [18]. It has been reported that processed and modified coal gangue can enhance adsorption capacity. For instance, Mohammadi et al. [19] used iron oxide to process coal gangue, achieving adsorption capacities of 42.10 mg/g and 30.12 mg/g for Zn(II) and Mn(II), respectively. Wenting Z. et al. [17] prepared a porous silicate adsorbent from coal gangue, with adsorption capacities of 168.92 mg/g and 234.19 mg/g for Cd(II) and methylene blue, respectively. Haodong L. et al. [20] synthesized an adsorbent by mixing calcined coal gangue with peanut shells, achieving an adsorption capacity of 0.954 mg/g for methylene blue. Zhou et al. [21] used calcined coal gangue to form ceramic microspheres, with adsorption capacities of 1.04 mg/g and 2.17 mg/g for cationic red and cationic blue dyes, respectively. Wenting Z. et al. [22] synthesized a novel porous heterogeneous composite material from calcined coal gangue, with adsorption capacities of 269.86 mg/g and 504.91 mg/g for methylene blue and basic red 14, respectively. These studies suggest that processed and modified coal gangue can serve as an adsorbent for dye adsorption. However, research on combining HAP with coal gangue for dye adsorption remains relatively limited.

Therefore, in this study, coal gangue, along with CaCl_2_, (NH_4_)_2_HPO_4_, and NH_3_·H_2_O, was used as raw materials to prepare a novel adsorbent, coal gangue-loaded hydroxyapatite (CG@HAP), based on the chemical precipitation method. CG@HAP was then applied in the treatment of malachite green dye wastewater. Static batch experiments were conducted to investigate the effects of pH, CG@HAP dosage, adsorption time, and malachite green concentration on the adsorption of malachite green by CG@HAP. Dynamic tests were also carried out to analyze the persistence of CG@HAP in the dynamic adsorption of malachite green dye. Using adsorption kinetics and isotherm models, combined with XRF, FTIR, XRD, and SEM analyses, the mechanism of CG@HAP adsorption of malachite green was revealed. The results demonstrate the feasibility of converting coal gangue from solid waste into an environmental purification material, promoting sustainable recycling.

## 2. Results and Discussion

### 2.1. Effect of pH on the Adsorption of Malachite Green by CG@HAP

**Figure 1:**
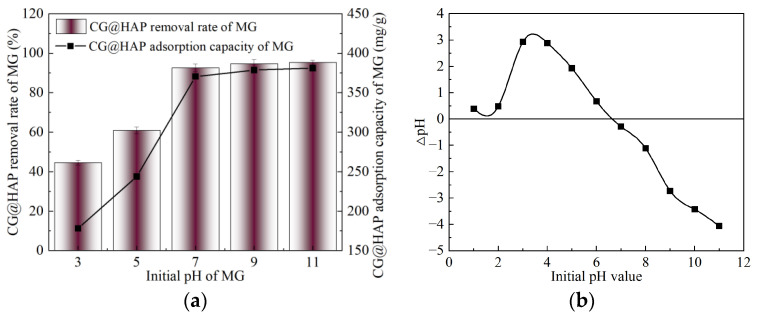
The effect of pH on the adsorption of malachite green by CG@HAP. (**a**) The effect of CG@HAP on the adsorption of malachite green under different pH conditions. (**b**) The point of zero charge (pH_PZC_) distribution of CG@HAP.

As shown in Figure 1a, the pH of the malachite green solution affects the adsorption of malachite green by CG@HAP. When the pH is in the range of 3 to 7, the removal rate of malachite green by CG@HAP increases with the rise in pH, from 44.52% to 92.62%. When the pH is in the range of 7 to 11, the removal rate stabilizes as the pH increases further, maintaining a range of 92.62% to 95.27%. This indicates that an acidic environment is not conducive to the adsorption of malachite green by CG@HAP, while an alkaline environment favors the adsorption process. These results can be explained by analyzing the surface charge of malachite green and the surface charge of the adsorbent CG@HAP before and after the adsorption process. The point of zero charge (pH_PZC_) of CG@HAP is shown in Figure 1b. At pH = pH_PZC_, the surface of CG@HAP is neutral. When the pH is greater than pH_PZC_, CG@HAP carries a negative charge, and when the pH is less than pH_PZC_, CG@HAP carries a positive charge. As shown in Figure 1b, the pH_PZC_ of CG@HAP is 6.6. This means that when the pH is below 6.6, the adsorption sites on CG@HAP are positively charged, which, due to electrostatic repulsion, hinders the adsorption of the positively charged malachite green. When the pH is above 6.6, the surface of CG@HAP carries a negative charge, which allows it to adsorb positively charged malachite green through electrostatic attraction, resulting in a higher adsorption capacity. Therefore, due to electrostatic interactions, a mildly alkaline environment is beneficial for the adsorption of malachite green by CG@HAP.

### 2.2. Effect of CG@HAP Dosage, Adsorption Time, Malachite Green Concentration, and Coexisting Dyes on the Adsorption of Malachite Green by CG@HAP

**Figure 2:**
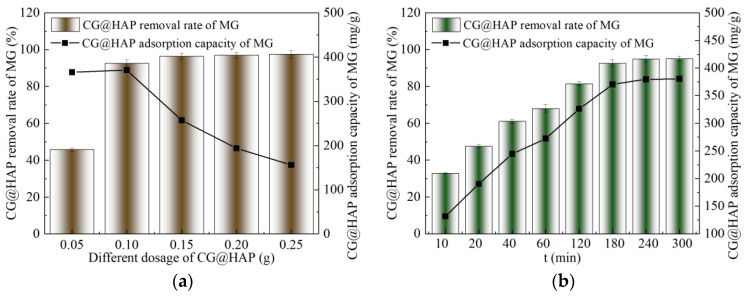

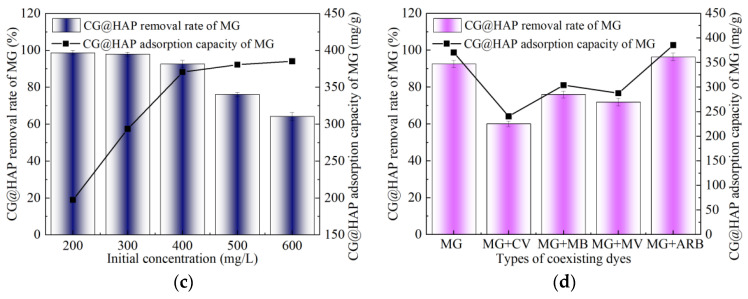
Effect of different factors on the adsorption of malachite green by CG@HAP. (**a**) Effect of CG@HAP dosage. (**b**) Effect of adsorption time. (**c**) Effect of malachite green concentration. (**d**) Effect of coexisting dyes.

As shown in Figure 2a, the adsorption removal rate of malachite green by CG@HAP initially increases and then tends to stabilize as the CG@HAP dosage increases. When 0.05 g of CG@HAP is added to 100 mL of malachite green solution, the removal rate of malachite green by CG@HAP is only 45.70%. When the CG@HAP dosage is increased to 0.10–0.25 g, the removal rate stabilizes between 92.62% and 97.49%. This indicates that when the CG@HAP dosage is in the range of 0.05–0.10 g, the dosage is the main factor influencing the adsorption of malachite green by CG@HAP. However, when the CG@HAP dosage exceeds 0.10 g, it no longer affects the adsorption efficiency. Additionally, Figure 2a shows that as the CG@HAP dosage increases from 0.05 g to 0.10 g, the adsorption capacity of CG@HAP increases from 365.58 mg/g to 370.49 mg/g. However, when the CG@HAP dosage is in the range of 0.10–0.25 g, the adsorption capacity decreases from 370.49 mg/g to 155.99 mg/g as the dosage increases. This indicates that the amount of malachite green in the solution is limited, and when the CG@HAP dosage is too high, nearly all the malachite green in the solution is removed. At this point, there are still excess binding sites on CG@HAP, leading to a waste of the adsorbent. Therefore, when the CG@HAP dosage is 0.10 g, the adsorption of malachite green by CG@HAP is optimal, while also conserving the amount of CG@HAP used.

As shown in Figure 2b, with the extension of adsorption time, the removal rate and adsorption capacity of malachite green by CG@HAP initially increase and then stabilize. At an adsorption time of 180 min, the removal rate and adsorption capacity reach their maximum values, 92.62% and 370.49 mg/g, respectively.

As shown in Figure 2c, the adsorption rate of CG@HAP for malachite green generally decreases with an increase in malachite green concentration. The maximum removal rate of 98.67% is achieved when the initial concentration of malachite green is 200 mg/L. When the initial concentration is 600 mg/L, the removal rate drops to a minimum of 64.19%. However, the adsorption capacity of CG@HAP increases with an increase in malachite green concentration. At an initial concentration of 200 mg/L, the adsorption capacity is the lowest, at 197.34 mg/g. When the initial concentration is in the range of 400–600 mg/L, the adsorption capacity stabilizes between 370.49 and 385.11 mg/g. This is because, when the CG@HAP dosage remains constant, the adsorption of malachite green by CG@HAP transitions from unsaturated to saturated as the concentration of malachite green increases.

It can be seen from Figure 2d that the coexisting dyes significantly affect the adsorption rate of CG@HAP for malachite green, compared with CG@HAP adsorbing single malachite green solution. In the mixed solutions of malachite green and crystal violet (CV), malachite green and methylene blue (MB), malachite green and methyl violet (MV), the adsorption rate of malachite green by CG@HAP decreased significantly. The removal rate of malachite green decreased by 32.48%, 16.60% and 20.70%, respectively. This shows that crystal violet, methylene blue, methyl violet, and malachite green exhibit competitive adsorption when CG@HAP is used as an adsorbent for dyes. However, compared with malachite green, the adsorption rate of CG@HAP to malachite green in malachite green + acid red B (ARB) solution increased by 3.76%, reaching 96.38%. This shows that a promotive adsorption relationship between acid red B and malachite green when CG@HAP is used as the adsorbent. The main reason for this difference in adsorption is that malachite green, crystal violet, methylene blue, and methyl violet dyes are cationic dyes, and they are positively charged after ionization, while acid red B is an acid dye, which is negatively charged after ionization. It can be seen from Figure 1b that when the pH value is above 6.6, the surface of CG@HAP is negatively charged, and the positively charged dyes can be adsorbed by electrostatic attraction. Therefore, malachite green, crystal violet, methylene blue, and methyl violet dyes are competitively adsorbed on the surface of adsorbent CG@HAP. Among them, crystal violet dye has the greatest influence on the adsorption of malachite green by CG@HAP.

### 2.3. Adsorption Kinetics of Malachite Green by CG@HAP

**Figure 3:**
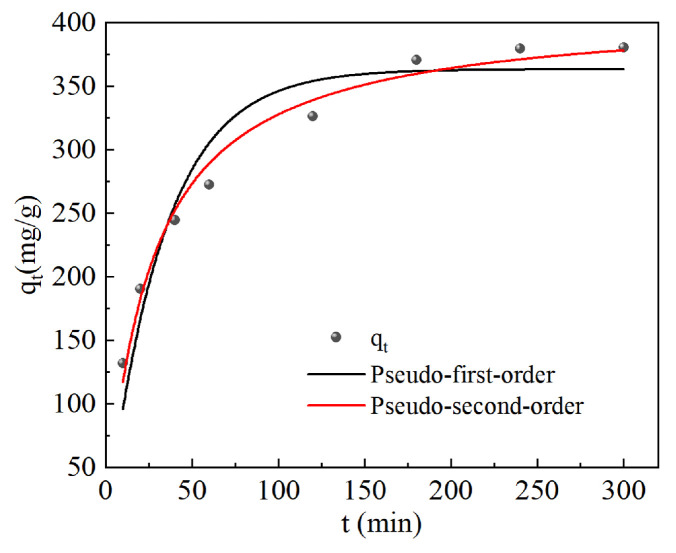
Adsorption kinetics fitting curves of malachite green by CG@HAP.

The adsorption kinetics fitting results of malachite green by CG@HAP are shown in Figure 3 and Table 1.

As shown in Figure 3 and Table 1, the *R*^2^ value for the pseudo-second-order kinetic model of CG@HAP’s adsorption of malachite green is greater than that for the pseudo-first-order model. This indicates that the adsorption process of malachite green by CG@HAP follows the pseudo-second-order kinetic model. The adsorption kinetic equation of CG@HAP for malachite green is *q_t_* = 16.38*t*/(1 + 0.03996*t*), *R*^2^ = 0.984. The above results suggest that the adsorption of malachite green by CG@HAP is primarily governed by chemical reactions.

### 2.4. Adsorption Isotherms of Malachite Green by CG@HAP

**Figure 4:**
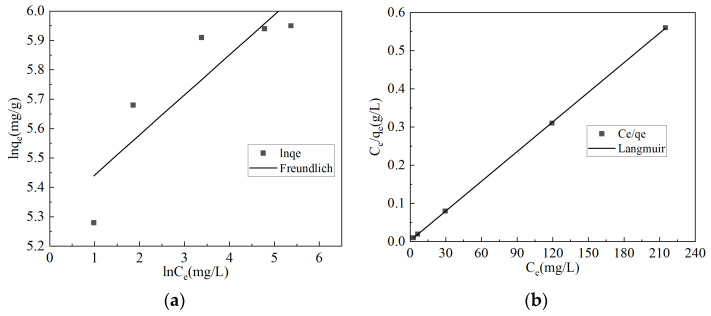
Freundlich and Langmuir isotherm fitting curves for the adsorption of malachite green by CG@HAP. (**a**) Freundlich isotherm fitting curves for the adsorption of malachite green by CG@HAP. (**b**) Langmuir isotherm fitting curves for the adsorption of malachite green by CG@HAP.

The adsorption isotherm fitting results of malachite green by CG@HAP are shown in Figure 4 and Table 2.

As shown in Figure 4 and Table 2, the *R*^2^ value for the Langmuir model fitting of malachite green adsorption by CG@HAP is greater than that for the Freundlich model fitting. This indicates that the adsorption process of malachite green by CG@HAP is more consistent with the Langmuir model. It also suggests that the adsorption of malachite green on CG@HAP follows the Langmuir monolayer adsorption theory, indicating that the distribution of adsorption sites on the CG@HAP surface is uniform and energy equivalent. This is primarily attributed to CG@HAP’s ability to reduce the agglomeration of HAP, improving the uniform dispersion of HAP crystals. The high surface activity of HAP crystals endows CG@HAP with efficient and uniform adsorption characteristics. According to the Langmuir model for the adsorption of malachite green by CG@HAP, the saturated adsorption capacity of CG@HAP for malachite green is 386 mg/g. By calculating the adsorption equilibrium constant (*R_L_*) (Equation (1)), it can be seen that (0 < *R_L_* < 1), indicating that the adsorption process of malachite green by CG@HAP is favorable [23].

(1) $$R_{L}=\frac{1}{1+C_{0}K_{L}}$$

### 2.5. Dynamic Adsorption Performance of CG@HAP for Malachite Green

**Figure 5:**
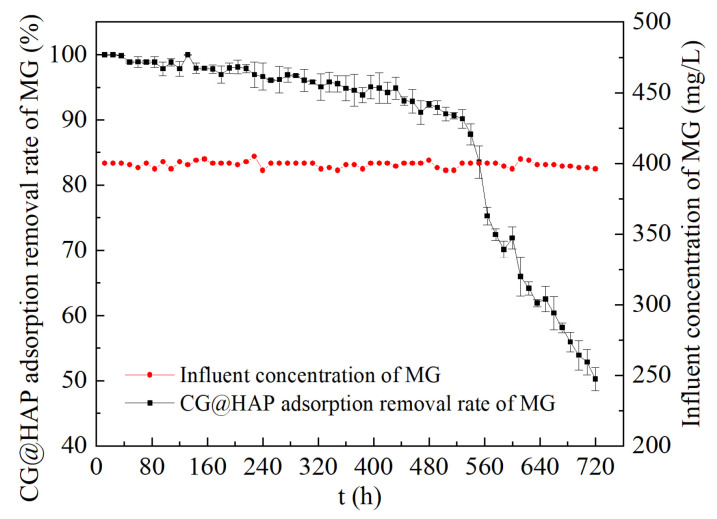
Dynamic adsorption performance of CG@HAP for malachite green.

As shown in Figure 5, the dynamic experimental results of CG@HAP’s adsorption of malachite green can be divided into two stages. The first stage, from 0 to 552 h, is characterized by rapid adsorption of malachite green. During this phase, there are many effective adsorption sites on the surface of CG@HAP, allowing for the quick and efficient adsorption of malachite green. The strong adsorption capacity of coal gangue, the reactivity of HAP, and the strong electrostatic attraction contribute to a stable removal rate of malachite green by CG@HAP, ranging from 83.52% to 99.96%. The second stage, from 552 to 720 h, represents a slow adsorption phase. At this point, the effective adsorption sites on the surface of CG@HAP gradually become filled with malachite green, and the adsorption starts to approach saturation. In this stage, the removal rate of malachite green by CG@HAP declines significantly, ultimately dropping to 50.26%. In summary, CG@HAP can continuously and efficiently adsorb malachite green dynamically. This provides a technical reference for the sustained remediation of malachite green dye wastewater.

### 2.6. XRF, FTIR, XRD, SEM, XPS, and BET Analysis

As shown in Table 3, the main components of CG@HAP are similar to those of coal gangue. However, compared to coal gangue, the content of Ca and P in CG@HAP has significantly increased. This indicates that HAP, which is primarily composed of Ca and P, has been successfully loaded onto the surface of coal gangue.

**Figure 6:**
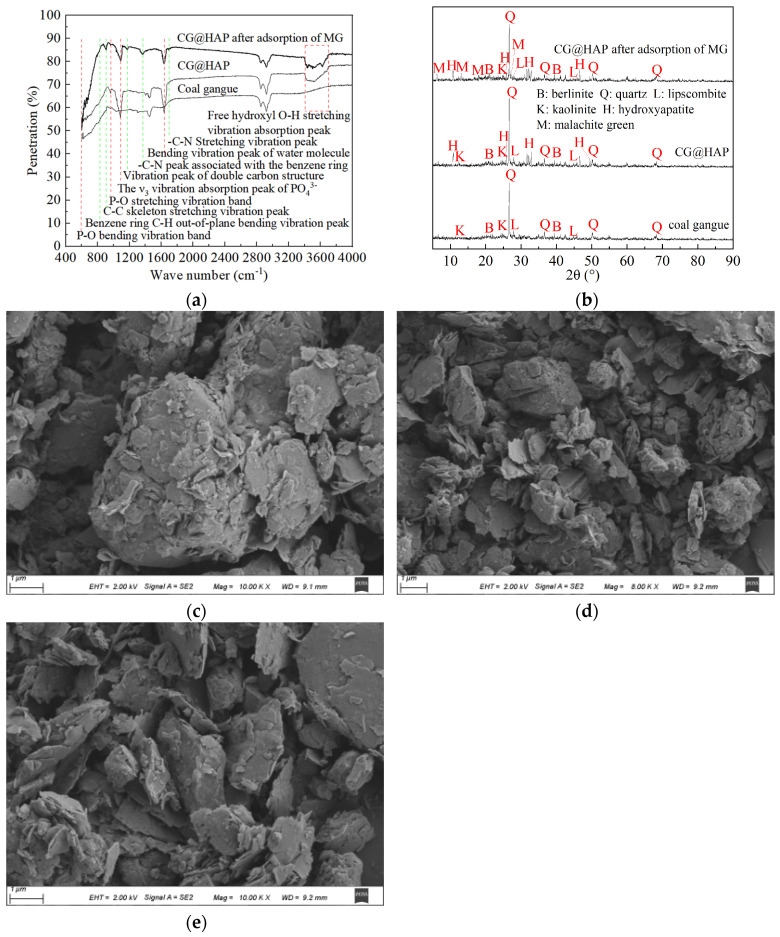
FTIR, XRD, and SEM images of CG@HAP adsorbing malachite green. (**a**) FTIR of CG@HAP adsorbing malachite green. (**b**) XRD of CG@HAP adsorbing malachite green. (**c**) SEM of coal gangue. (**d**) SEM of CG@HAP. (**e**) SEM of CG@HAP after adsorbing malachite green.

As shown in Figure 6a, compared to coal gangue, CG@HAP exhibits a P-O bending vibration band at 603.61 cm^−1^, a P-O stretching vibration band at 964.23 cm^−1^, and a ν_3_ vibration absorption peak for PO_4_^3−^ at 1089.58 cm^−1^, indicating the presence of PO_4_^3−^ in the CG@HAP sample [24,25,26]. A bending vibration peak for water molecules appears at 1637.27 cm^−1^, and the absorption peaks at 3400–3700 cm^−1^ correspond to the stretching vibration of free hydroxyl O-H. The presence of these absorption peaks confirms that HAP has been successfully loaded onto coal gangue. Overall, the prepared CG@HAP sample contains a significant amount of PO_4_^3−^ and OH^−^, which are beneficial for enhancing the adsorption of malachite green. When CG@HAP adsorbs malachite green, characteristic peaks for the dye appear at 829.24 cm^−1^, 908.31 cm^−1^, 1174.44 cm^−1^, 1369.21 cm^−1^, and 1695.12 cm^−1^, corresponding to out-of-plane bending vibrations of the benzene ring C-H, C-C skeletal stretching vibrations, vibrations of the bi-carbon structure, -C-N stretching vibrations connected to the benzene ring, and -C-N stretching vibrations, respectively. The emergence of these new absorption peaks indicates that CG@HAP can effectively adsorb malachite green. Notably, the PO_4_^3−^ and free hydroxyl absorption peaks for the adsorbed malachite green on CG@HAP at 603.61 cm^−1^, 964.23 cm^−1^, 1089.58 cm^−1^, and 3400–3700 cm^−1^ show changes, suggesting that PO_4_^3−^ and hydroxyl groups participate in the adsorption of malachite green. Combined with the pH_PZC_ of CG@HAP, it can be seen that functional groups such as PO_4_^3−^ and hydroxyl groups make the surface of CG@HAP negatively charged. It has been reported that HAP undergoes strong deprotonation to form functional groups such as ≡PO^—^, -(CaO)_2_-POO^—^, and ≡CaOH, which promotes the negative charge of apatite [25]. Functional groups such as PO_4_^3−^ and hydroxyl groups can use electrostatic attraction to adsorb positively charged malachite green, to achieve the effect of efficient CG@HAP adsorption of malachite green.

As shown in Figure 6b, coal gangue primarily contains characteristic diffraction peaks of quartz, lipscombite, berlinite, and kaolinite. After loading HAP onto coal gangue, in addition to the characteristic diffraction peaks of coal gangue, several new diffraction peaks appear in CG@HAP. Notably, CG@HAP exhibits characteristic diffraction peaks of HAP around 10.82°, 25.88°, 31.74°, 32.18°, 32.87°, 46.66°, and 49.46°. The HAP characteristic diffraction peaks at 10.82°, 25.88°, 32.87°, and 49.46° are significantly weakened after CG@HAP adsorbs malachite green. The diffraction peaks of kaolinite and lipscombite in coal gangue also show noticeable reductions. At the same time, CG@HAP adsorbed malachite green and showed new characteristic diffraction peaks of malachite green at 6.20°, 13.00°, 18.06°, and 27.21°, respectively. This indicates that HAP, kaolinite, and lipscombite are involved in the chemical reaction of CG@HAP adsorption of malachite green. Some scholars have also found similar conclusions in their research. For example, the magnesium-doped hydroxyapatite nanofibers prepared by Alaa T. Okasha et al. [25] can react with malachite green through electrostatic attraction, hydrogen bonding, π-π stacking, Lewis acid–base interaction, and van der Waals forces to fix malachite green in solution. The magnesium-doped hydroxyapatite nanofibers prepared by Guo et al. [26] could absorb malachite green. Wen X. et al. [27] reported that immobilized laccase from kaolin could effectively treat malachite green wastewater. The removal efficiency of malachite green by kaolin supported green synthetic nano-iron prepared by Cai et al. [28] can reach 99.10%, which was obviously better than that of green synthetic nano-iron material alone. Wang Yihan et al. [5] reported that lipscombite and dolomite are the key to the adsorption of malachite green by calcium-based modified coal gangue. The above reports also verified that HAP, kaolinite, and lipscombite were involved in the chemical reaction of malachite green adsorption.

The surface of coal gangue is relatively flat and smooth (Figure 6c). From Figure 6c,d, it can be observed that the surface of CG@HAP has many particulate materials. Based on the XRF, FTIR, and XRD results, these particles are the HAP loaded onto CG@HAP. The scale particles of 0.5–2 μm are clearly visible on the surface of CG@HAP, especially the scale particles of 0.5–1 μm. From Figure 6d,e, it can be seen that after the adsorption of malachite green by CG@HAP, the scale-like small particles loaded on the surface of CG@HAP decreased significantly, and only the scale-like large particles were left, with a length of about 1–2 μm. This indicates that the uniformly distributed HAP on the surface of coal gangue can easily adsorb malachite green, whereas the aggregated larger HAP particles have more difficulty in adsorbing the dye.

**Figure 7:**
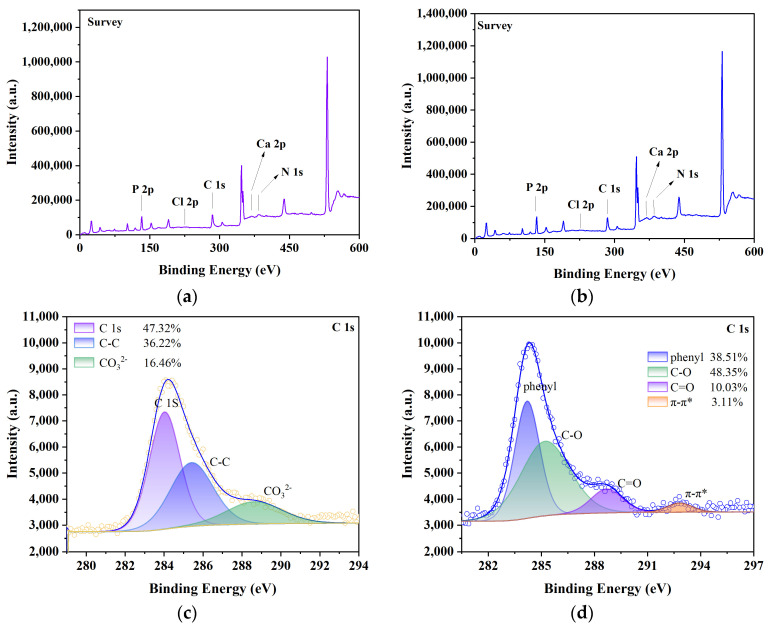

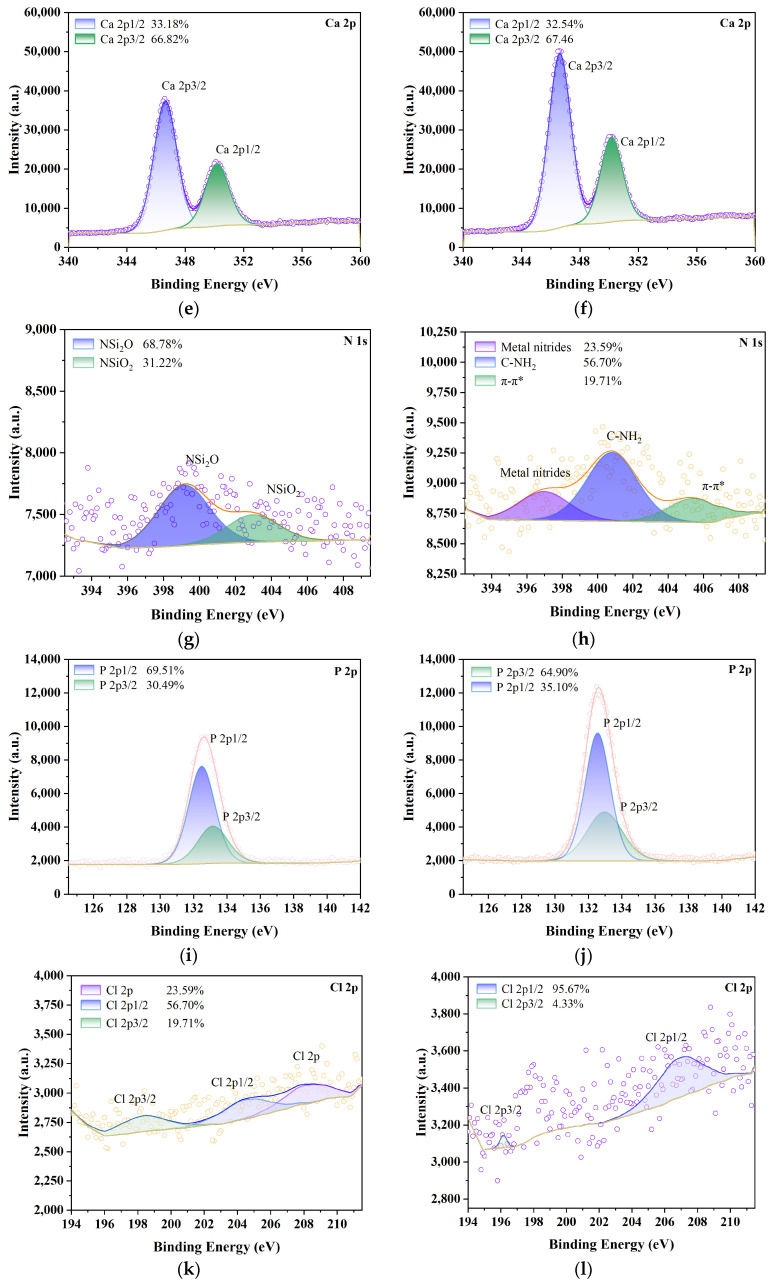

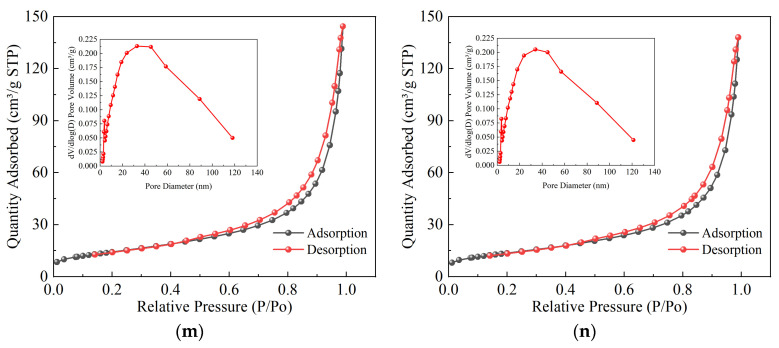
XPS and BET images of CG@HAP adsorbing malachite green. (**a**) The XPS detection map of CG@HAP. (**b**) The XPS detection map of CG@HAP after adsorption of malachite green. (**c**) C fine map in XPS of CG@HAP. (**d**) C fine map in XPS of CG@HAP after adsorption of malachite green. (**e**) XPS Ca fine map of CG@HAP. (**f**) XPS Ca fine map of CG@HAP after adsorption of malachite green. (**g**) N fine map in XPS of CG@HAP. (**h**) N fine map in XPS of CG@HAP after adsorption of malachite green. (**i**) XPS P fine map of CG@HAP. (**j**) XPS P fine map of CG@HAP after adsorption of malachite green. (**k**) Cl fine map in XPS of CG@HAP. (**l**) Cl fine map in XPS of CG@HAP after adsorption of malachite green. (**m**) BET of CG@HAP. (**n**) BET of CG@HAP after adsorption of malachite green.

The XPS results of CG@HAP before and after adsorption of malachite green are shown in Figure 7a–l. It can be seen from Figure 7a that the contents of C1s, Ca2p, N1s, P2p, and Cl2p in CG@HAP were 32.06%, 33.76%, 3.36%, 29.18%, and 1.64%, respectively. It can be seen from Figure 7b that the contents of C1s, Ca2p, N1s, P2p, and Cl2p in CG@HAP adsorbed malachite green were 28.43%, 33.57%, 2.20%, 27.92%, and 7.89%, respectively. The content of Ca2p and P2p in CG@HAP decreased by 0.19% and 1.26%, respectively, after adsorption of malachite green, which indicated that HAP was consumed during adsorption of malachite green and played a key role in adsorption. The content of Cl2p increased by 6.25%, mainly because malachite green contained Cl, and CG@HAP adsorbed a large amount of malachite green, resulting in an increase in Cl content. From Figure 7c, it can be seen that the C in CG@HAP mainly exists in the form of CO_3_^2−^, C-C, and C1s, with contents of 16.46%, 36.22%, and 47.32%, respectively. From Figure 7d, it can be seen that after the reaction, C mainly exists in the form of π-π* (satellite), C=O, C-O, and C1s (phenyl), with contents of 3.11%, 10.03%, 48.35%, and 38.51%, respectively. Among them, the π-π* (satellite) structure indicates an aromatic ring. After the adsorption of malachite green by CG@HAP, a large number of new organic structures appeared in the C component, which indicated that malachite green was adsorbed on the surface of CG@HAP. It can be seen from Figure 7e,f that Ca mainly exists in the form of Ca2p_1/2_ and Ca2p_3/2_ before and after adsorption. It can be seen from Figure 7g that the N in CG@HAP mainly exists in the form of NSiO_2_ and NSi2O, and the contents are 31.22% and 68.78%, respectively. From Figure 7h, it can be seen that after the reaction, N mainly exists in the form of π-π* (satellite), C-NH_2_, and metal nitrides, with contents of 19.71%, 56.70%, and 23.59%, respectively. The π-π* (satellite) structure indicates the presence of nitrogen-containing aromatic polymers. After the adsorption of malachite green by CG@HAP, a large number of nitrogen-containing aromatic polymers and C-N appeared in the N component, indicating that malachite green was adsorbed on the surface of CG@HAP. From Figure 7i,j, it can be seen that P before and after adsorption mainly exists in the form of P2p_1/2_ (phosphate) and P2p_3/2_ (phosphate). It can be seen from Figure 7k that Cl in CG@HAP mainly exists in the form of Cl2p, Cl2p_1/2_ (chloride), and Cl2p_3/2_ (chloride). It can be seen from Figure 7l that Cl in CG@HAP mainly exists in the form of Cl2p_1/2_ (chloride) and Cl2p_3/2_ (chloride). In summary, after the adsorption of malachite green by CG@HAP, new aromatic rings, phenyl, nitrogen-containing aromatic polymers, C-N, etc. appeared, indicating that CG@HAP can effectively adsorb and fix malachite green dye.

The BET test results of CG@HAP before and after adsorption of malachite green are shown in Figure 7m,n and Table 4. The surface area of CG@HAP before and after adsorption of malachite green was 51.6 m^2^/g and 49.2 m^2^/g, respectively. The surface area of CG@HAP decreased by 4.65% after adsorption of malachite green. This is mainly because during the adsorption process, CG@HAP adsorbs malachite green, which occupies part of the surface area, leading to a decrease in the specific surface area. The pore volume of CG@HAP before and after adsorption of malachite green was 0.223 cm^3^/g and 0.214 cm^3^/g, respectively. The material exchange during the adsorption process led to the occupation of some pores, so that the pore volume of CG@HAP decreased by 4.04% after adsorbing malachite green. The pore size of CG@HAP before and after adsorption of malachite green was 17.3 nm and 17.4 nm, respectively. The pore size of CG@HAP increased by 0.58% after adsorption of malachite green, which was due to the fact that malachite green filled part of the micropores of the adsorbent, reducing the number of micropores and increasing the proportion of mesopores.

### 2.7. Discussion

The new adsorbent CG@HAP prepared in this study has a saturated adsorption capacity of 386 mg/g for malachite green. As shown in Table 5, compared to the HAP conjugated graphene oxide nanocomposite [29], the CG@HAP in this study exhibits a significantly higher adsorption capacity for malachite green, approximately 2.16 times greater. This indicates that the adsorbent formed by the combination of HAP and coal gangue is more effective in adsorbing malachite green than the adsorbent formed by the combination of HAP and graphene oxide. Compared to other adsorbents for malachite green [30,31,32,33,34,35,36,37,38], the CG@HAP in this study also demonstrates a strong adsorption effect. Particularly, the adsorption capacity of CG@HAP for malachite green is dozens of times higher compared to magnetite/coir pith-supported sodium alginate beads [30], Fe_3_O_4_@chitosan@ZIF-8 [31], activated lupinus albus seed peel waste [32], Mn-Fe layered double hydroxide/polyethersulfone composite [33], and Chinese Fan-Palm biochar [34]. Therefore, the newly developed adsorbent, CG@HAP, has a strong ability to adsorb malachite green. As shown in Table 5, HAP or HAP composites [39,40,41,42,43,44,45,46] can effectively adsorb dyes such as reactive blue, methylene blue, congo red, and acid yellow. Compared to Chitosan-coated magnetic HAP, Calcined nanohydroxyapatite, Uncalcined nanohydroxyapatite, HAP, Microwave-HAP, Magnetic HAP, HAP/chitosan composite, Polyalcohol-coated HAP, Mg HAP, and Sodium alginate HAP, the CG@HAP prepared in this study still exhibits strong organic dye adsorption capacity. This fully demonstrates that the newly developed adsorbent CG@HAP can efficiently adsorb organic dyes. In the future, the optimization and improvement of the CG@HAP adsorbent and its application in other wastewater fields need to be further explored. For example, CG@HAP is combined with metal–organic frameworks (MOFs) [47], magnetic covalent organic frameworks [48], and other materials. MOFs are a class of porous inorganic–organic hybrid networks synthesized by multidentate organic ligands [49,50]. The magnetic covalent organic framework is a new type of magnetic porous material, which is composed of organic molecules connected by covalent bonds [48]. The combination of CG@HAP and these skeletons can not only effectively expand the treatment of different contaminated wastewater by adsorbent CG@HAP but also improve the adsorption effect of CG@HAP, which is convenient for recycling.

In this study, it was found that crystal violet, methylene blue, methyl violet, and malachite green were competitive when CG@HAP was used as adsorbent to adsorb dyes. Under the same experimental conditions, the order of the three dyes affecting the adsorption of malachite green by CG@HAP is: crystal violet > methyl violet > methylene blue. However, when CG@HAP is used as an adsorbent, acid red B dye and malachite green dye form the relationship that promotes adsorption. This is mainly because malachite green, crystal violet, methylene blue, and methyl violet dyes are positively charged after ionization. Acid red B is negatively charged after ionization. The surface of CG@HAP was negatively charged under the initial pH condition of the mixed dye. Therefore, due to the electrostatic attraction, malachite green, crystal violet, methylene blue and methyl violet dyes are competitive adsorption on CG@HAP. T.S. Anirudhan et al. [51] synthesized a polyacrylamide/bentonite composite material, and its order of dye adsorption capacity was as follows: malachite green > methylene blue > crystal violet. Yihan Wang et al. [5] synthesized a calcium-based modified coal gangue, and its adsorption effect on dyes was as follows: malachite green > crystal violet > methylene blue > methyl violet. Jun Wei Fan et al. [52] used polyacrylonitrile and calcium lignosulfonate as raw materials to prepare composite membranes. The adsorption effects of the composite membranes on dyes were as follows: methylene blue > congo red. Wenjuan Wu et al. [53] synthesized a lignosulfonate/polyaniline nanocomposite adsorption material. The order of dye adsorption capacity was as follows: malachite green > methylene blue > crystal violet. The above studies show that the adsorbent will show different adsorption effects on different dyes. The above adsorbents showed good adsorption effect on malachite green. Interestingly, CG@HAP prepared in this study still showed a good adsorption effect on malachite green under the condition of mixed dyes. This is similar to the adsorption properties of other adsorption materials. This shows that the adsorbent CG@HAP can be applied in the field of dye wastewater treatment such as malachite green.

## 3. Materials and Methods

### 3.1. Test Materials

The CaCl_2_, (NH_4_)_2_HPO_4_, and NH_3_·H_2_O used in the experiment were all sourced from Tianjin Dengfeng Chemical Reagent Factory (Tianjin, China). The malachite green and other dyes are from Tianjin Fuchen Chemical Reagent Co., Ltd. (Tianjin, China). The main instruments used in the experiment included an ultrapure water machine (YL-400BU model, Shenzhen EREERAN Water Treatment Equipment Co., Ltd., Guangdong, China), a constant temperature magnetic stirrer (HJ-1 model, Changzhou Surui Instrument Co., Ltd., Jiangsu, China), a forced air drying oven (GZX-9246MBE model, Shanghai Boxun Medical Biological Instrument Co., Ltd., Shanghai, China), and a spectrophotometer (721 model, Shanghai Jinghe Analytical Instrument Co., Ltd., Shanghai, China).

Preparation of CG@HAP was as follows: 10 g of coal gangue and 150 mL of 0.25 mol/L CaCl_2_ solution were added to a beaker. NH_3_·H_2_O was used to adjust the pH of the solution in the beaker to 10. The mixture was stirred in a constant temperature water bath at 60 °C. During stirring, 150 mL of 0.15 mol/L (NH_4_)_2_HPO_4_ solution was slowly added dropwise, while NH_3_·H_2_O was continuously added to maintain the pH at 10. The mixture was left to age at room temperature for 24 h. After aging, the mixture was filtered and washed several times with deionized water, followed by drying at 60 °C. The material was then sieved to 80 mesh to obtain CG@HAP.

### 3.2. Test Methods

To investigate the adsorption effect of CG@HAP on malachite green, a series of batch adsorption experiments were conducted (Table 6) to explore the influence of pH, CG@HAP dosage, adsorption time, and malachite green concentration on the adsorption of malachite green by CG@HAP. The specific steps were as follows: malachite green solutions of a certain concentration were prepared, and the pH of the malachite green solution was adjusted using 0.1 mol/L HCl and NaOH solutions. 100 mL of the malachite green solution was poured into a 250 mL conical flask, and a certain mass of CG@HAP was added. The mixture was shaken at 150 rpm and 25 °C for a set time. After adsorption, the solution was filtered using a 0.22 μm filter membrane (Haining Chuangwei filter equipment factory, Zhejiang, China). The filtrate was taken, and the remaining concentration of malachite green was measured using a spectrophotometer (Model 721, Shanghai Jinghe Analytical Instrument Co., Ltd., Shanghai, China) at a wavelength of 617 nm. The adsorption capacity, *q_e_* (Equation (2)), and adsorption rate, η (Equation (3)), were calculated. Detailed parameters for the batch adsorption experiments are provided in Table 6. After the batch adsorption test, the effect of coexisting dyes on the adsorption of malachite green by CG@HAP was tested. The specific steps were as follows: 0.1 g CG@HAP was added to 100 mL dye composite solution (400 mg/L malachite green and 400 mg/L crystal violet (CV), 400 mg/L malachite green and 400 mg/L methylene blue (MB), 400 mg/L malachite green and 400 mg/L methyl violet (MV), and 400 mg/L malachite green and 400 mg/L acid red B (ARB)). The concentration of malachite green was determined after shaking at 150 r/min and 25 °C for 180 min, and the removal rate of malachite green was calculated. Each experiment was repeated three times, and the average values were used to plot the graphs. Based on the results of the batch experiments, the Lagergren first-order kinetic equation (Equation (4)) and the Lagergren second-order kinetic equation (Equation (5)) were used to fit the results, exploring the adsorption kinetics of CG@HAP on malachite green. The Langmuir (Equation (6)) and Freundlich (Equation (7)) adsorption isotherm models were employed to investigate the adsorption isotherms of CG@HAP on malachite green.

(2) $$q_{e}=\frac{V\times (C_{0}-C_{e})}{m}$$

(3) $$\eta =\frac{C_{0}-C_{e}}{C_{0}}\times 100\%$$

(4) $$q_{t}=q_{e}\times (1-\exp(-k_{1}\times t))$$

(5) $$q_{t}=\frac{k_{2}\times q_{e}^{2}\times t}{1+k_{2}\times q_{e}\times t}$$

(6) $$\frac{C_{e}}{q_{e}}=\frac{1}{q_{m}K_{L}}+\frac{C_{e}}{q_{m}}$$

(7) $$lnq_{e}=lnK_{F}+\frac{1}{n}lnC_{e}$$

In the formulas: *q_e_* (mg/g) is the adsorption capacity of CG@HAP for malachite green. *V* (L) is the volume of the malachite green solution. *C*_0_ (mg/L) and *C_e_* (mg/L) are the initial and equilibrium concentrations of malachite green, respectively. *m* (g) is the mass of CG@HAP added. *η* (%) is the adsorption rate of CG@HAP for malachite green. *q_t_* (mg/g) is the adsorption capacity of CG@HAP for malachite green at time *t* (min); *k*_1_ (min^−1^) and *k*_2_ (mg/(g·min)) are the adsorption rate constants for the Lagergren first-order and second-order kinetic models, respectively. *q*_m_ (mg/g) is the maximum adsorption capacity of CG@HAP for malachite green at saturation; *K_L_* (L/mg) and *K_F_* (mg^(1−1/*n*)^·L^1/*n*^·g^−1^) are the adsorption constants for the Langmuir and Freundlich models, respectively; and *n* is the constant representing the adsorption intensity of CG@HAP for malachite green in the Freundlich model.

The dynamic experiment method for CG@HAP adsorption of malachite green is as follows: An organic glass tube with an inner diameter of 50 mm and a height of 500 mm is used as the dynamic reaction device. The device is filled with 50 mm of glass beads +300 mm of CG@HAP +50 mm of glass beads. The glass beads primarily prevent the loss of the adsorbent CG@HAP. A peristaltic pump is used to introduce a 400 mg/L malachite green solution into the bottom of the dynamic device at a flow rate of 10 mL/h. The malachite green solution flows through the CG@HAP and exits from the top of the dynamic device. This bottom-to-top flow method is mainly to expel air from the device, ensuring sufficient contact between CG@HAP and the malachite green solution. Water samples from the inlet and outlet are collected every 12 h to measure malachite green concentrations and calculate the removal rate.

The detection methods for XRF, FTIR, XRD, SEM, XPS, and BET are as follows: Samples of coal gangue, CG@HAP, and CG@HAP adsorbing malachite green are subjected to XRF, FTIR, XRD, and SEM analysis. The XRF analysis is conducted using an XRF-1800 instrument from Shimadzu, Japan (Kyoto, Japan). The XRD analysis is performed with a Smart Lab 9 instrument from Rigaku, Japan (Tokyo, Japan). FTIR detection is carried out using a Nicolet iS5 instrument from Thermo Fisher, USA (Waltham, MA, USA). SEM analysis is performed with a Sigma 500 instrument from Zeiss, Germany (Oberkochen, Germany). XPS detection was performed using the EscaLab 250 Xi instrument (Waltham, MA, USA). BET detection was performed using an American Mike ASAP 2020 instrument (Atlanta, GA, USA).

## 4. Conclusions

This study developed a novel adsorbent, CG@HAP, using coal gangue, CaCl_2_, (NH_4_)_2_HPO_4_, and NH_3_·H_2_O as raw materials. The prepared adsorbent, CG@HAP, demonstrates economic and efficient removal of malachite green dye. Additionally, CG@HAP provides a new pathway for the resource utilization of solid waste, specifically coal gangue. The characteristics of the prepared adsorbent CG@HAP are as follows:

(1) Acidic environments hinder the adsorption of malachite green by CG@HAP, while weakly alkaline conditions favor the adsorption. When the dosage of CG@HAP is 0.10 g and the adsorption time is 180 min, the removal rate and adsorption capacity of CG@HAP for a 400 mg/L malachite green solution reach 92.62% and 370.49 mg/g, respectively. As the concentration of malachite green increases, the removal rate by CG@HAP shows a decreasing trend. When CG@HAP is used as an adsorbent to adsorb dyes, crystal violet, methylene blue, methyl violet, and malachite green are competitive and acid red B dye and malachite green dye are promoting adsorption. The order of three dyes affecting the adsorption of malachite green by CG@HAP is as follows: crystal violet > methyl violet > methylene blue.

(2) The adsorption of malachite green by CG@HAP follows the second-order kinetic model and the Langmuir model. The adsorption is primarily driven by chemical reactions, conforming to the Langmuir monolayer adsorption theory. The maximum adsorption capacity of CG@HAP for malachite green is 386 mg/g.

(3) The dynamic adsorption process of CG@HAP for malachite green is divided into two stages: a rapid adsorption phase (0–552 h) and a slow adsorption phase (552–720 h). During the rapid adsorption phase, CG@HAP can continuously and efficiently adsorb malachite green, maintaining a removal rate between 83.52% and 99.96%.

(4) XRF, FTIR, XRD, and SEM analyses indicate that the particulate HAP is uniformly distributed on the surface of coal gangue. CG@HAP contains a significant amount of functional groups such as PO_4_^3−^ and hydroxyl groups, which impart a negative charge to the surface of CG@HAP, with a pHPZC of 6.6. The PO_4_^3−^ and hydroxyl functional groups on the surface of CG@HAP can utilize electrostatic attraction to adsorb positively charged malachite green. HAP, kaolinite, and lipscombite in CG@HAP participate in the chemical reaction for the adsorption of malachite green, achieving a high adsorption efficiency.

## Figures and Tables

**Table 1:** Kinetic fitting data for the adsorption of malachite green by CG@HAP.

Adsorption Kinetic Model	Parameters	Adsorption of Malachite Green by CG@HAP
Pseudo-first-order	*q*_e_ (mg/g)	363
	*k*_1_ (min^−1^)	0.0306
	*R* ^2^	0.926
Pseudo-second-order	*q*_e_ (mg/g)	409
	*k*_2_ (mg/(g·min))	0.000975
	*R* ^2^	0.984

**Table 2:** Freundlich and Langmuir isotherm fitting data for the adsorption of malachite green by CG@HAP.

Adsorption Isotherm Model	Parameters	Adsorption of Malachite Green by CG@HAP
Freundlich Model	*K_F_* (mg^(1−1/^*^n^*^)^·L^1/^*^n^*·g^−1^)	201
	*n*	7.34
	*R* ^2^	0.791
Langmuir Model	*q*_m_ (mg/g)	386
	*K_L_* (L/mg)	0.849
	*R* ^2^	0.999

**Table 3:** Main chemical components (%) of coal gangue and CG@HAP.

Component	SiO_2_	Al_2_O_3_	Fe_2_O_3_	MgO	K_2_O	CaO	Na_2_O	TiO_2_	MnO	P_2_O_5_	SO_3_	CO_2_	Other
Coal Gangue	60.81	18.47	4.98	2.95	2.19	1.87	1.54	0.81	0.08	2.35	0.28	2.56	1.11
CG@HAP	56.39	18.39	4.85	2.81	2.07	4.28	1.59	0.79	0.07	5.21	0.39	2.95	0.21

**Table 4:** BET Test data of CG@HAP before and after adsorption of malachite green.

Component	Surface Area (m^2^/g)	Pore Volume (cm^3^/g)	Pore Size (nm)
CG@HAP	51.6	0.223	17.3
CG@HAP after adsorption of malachite green	49.2	0.214	17.4

**Table 5:** Comparison of adsorption capacity of different adsorbents for dyes.

Adsorbates	Adsorbents	Q (mg/g)	Refs.
Malachite green	CG@HAP	386	this study
	HAP conjugated graphene oxide nanocomposite	178.5	[29]
	Magnetite/coir pith supported sodium alginate beads	1.67	[30]
	Fe_3_O_4_@chitosan@ZIF-8	3.28	[31]
	Activated lupinus albus seed peel waste	7.3	[32]
	Mn-Fe layered double hydroxide/polyethersulfone composite	13.49	[33]
	Chinese Fan-Palm biochar	21.4	[34]
	ZIF-8@Fe/Ni	151.52	[35]
	Organo-apatite	188.18	[36]
	Activated carbon	250	[37]
	Activated carbon loaded with ZnO nanoparticles	303.03	[38]
Reactive blue	Chitosan coated magnetic HAP	26	[39]
	Calcined nanohydroxyapatite	74.97	[40]
	Uncalcined nanohydroxyapatite	90.09	[40]
Methylene blue	HAP	14.3	[41]
	Microwave-HAP	33.3	[42]
	Magnetic HAP	328.4	[43]
Congo red	HAP/chitosan composite	769	[44]
	Polyalcohol-coated HAP	170.7	[45]
Acid yellow	Mg HAP	103.1	[46]
	HAP	169.5	[46]
	Sodium alginate HAP	212.8	[46]

**Table 6:** Batch experiment parameters for the adsorption of malachite green by CG@HAP.

Number	Experimental Factors	Experimental Conditions			
		pH Value of Malachite Green	Initial Concentration of Malachite Green (mg/L)	CG@HAP Dosage (g)	Adsorption Time (min)
1	pHvalue	3, 5, 7, 9, 11	400	0.1	180
2	CG@HAP dosage	7	400	0.05, 0.1,0.15, 0.20, 0.25	180
3	Adsorption time	7	400	0.1	10, 20, 40, 60, 120, 180, 240, 300
4	Concentration of malachite green	7	200, 300, 400, 500, 600	0.1	180

## Data Availability

All the data have been included in the study.
